# Rescue endoscopic treatment with completion by radical surgery following misplacement of a partially covered metal stent in an anastomotic fistula post-Lewis Santy esophagectomy

**DOI:** 10.1055/a-2371-0827

**Published:** 2024-08-07

**Authors:** Pierre Mayer, Lucile Héroin, François Habersetzer, Pierre-Yves Christmann, Jérôme Huppertz, Leonardo Sosa-Valencia, Abdenor Badaoui

**Affiliations:** 1 36604 Department of Gastroenterology and Hepatology, University Hospitals Strasbourg, Strasbourg, France; 2 560036 Digestive Endoscopy Unit, IHU Strasbourg, Strasbourg, France; 3 27083 Inserm U1110, Université de Strasbourg, Strasbourg, France; 4 Department of Hepatogastroenterology, Clinique Sainte Barbe, Strasbourg, France; 5 560036 Research, IHU Strasbourg, Strasbourg, France; 6 82470 Department of Gastroenterology and Hepatology, CHU UCL Namur, Yvoir, Belgium

Curative management of esophageal adenocarcinoma is based on esophagectomy. One of the main complications is anastomotic fistula (30%) 1 , which is responsible for significant postoperative morbidity and mortality, as well as reduced survival 2 . In recent years, endoscopic treatment of anastomotic fistulas has become a valuable option, enabling closure of the fistula and a reduction in the mortality rate 3 4 .

We report the case of a 55-year-old patient who underwent a Lewis Santy esophagectomy for esophageal adenocarcinoma. The patient developed an anastomotic fistula with a pleural abscess requiring antibiotics, thoracic drainage, and placement of a partially covered self-expandable metal stent (PCSEMS) to cover the fistula. However, the thoracic drainage remained highly productive and an endoscopy revealed migration of the stent’s distal flange, with embedment into the fistula ( Fig. 1 and Fig. 2 ). After several unsuccessful attempts at endoscopic removal, the patient was transferred to our center.

**Fig. 1:**
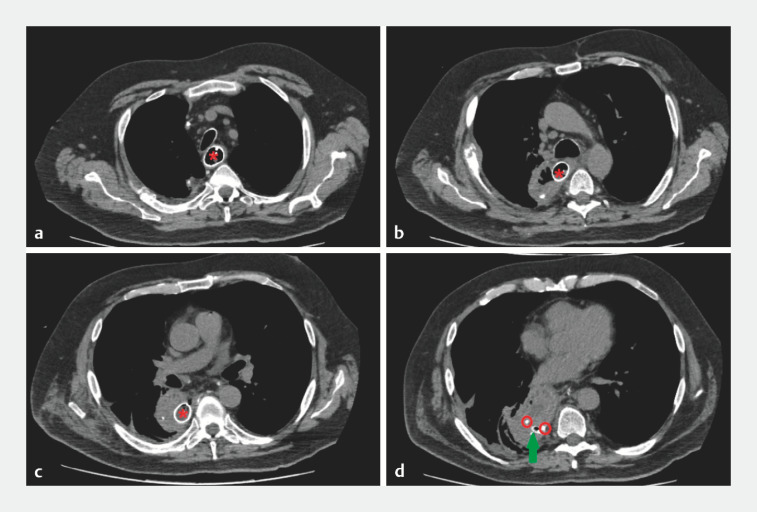
Computed tomography images from the initial scan showing: **a** the partially covered self-expandable metal stent (PCSEMS; red star) within the esophageal lumen; **b** the PCSEMS passing into the mediastinal cavity through the anastomotic fistula; **c** the PCSEMS within the mediastinal cavity, with a fluid and air-containing collection in this area; **d** the distal flange of the stent (red circles) in contact with the thoracic drain (green arrow).

**Fig. 2:**
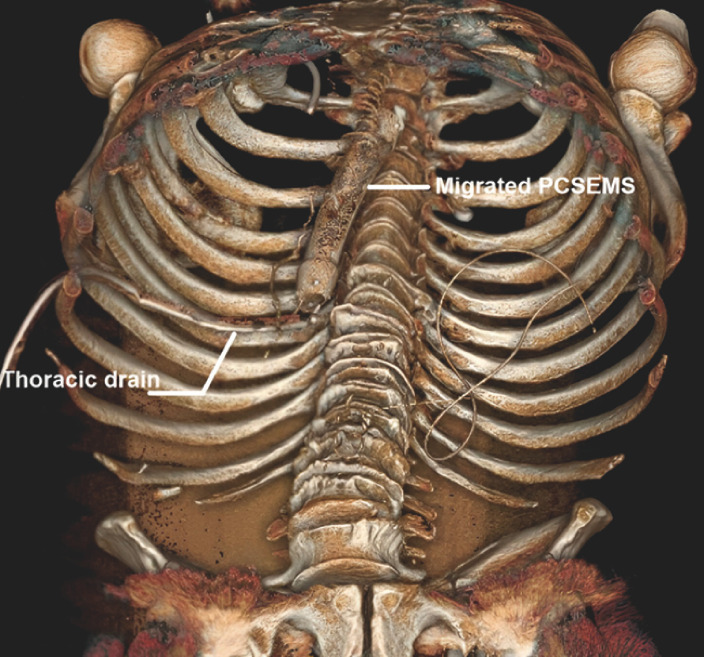
3D reconstruction from the initial computed tomography scan showing that the stent has migrated into the mediastinal cavity through the anastomotic fistula orifice.

He presented to us with a chronic pleural infection and total dependence on parenteral nutrition. An endoscopic procedure to re-establish digestive continuity was planned. The lower pole of the fibrin-wrapped stent and a productive fistulous orifice were identified ( Fig. 3 ). We managed to pass the scope in parallel to the stent to gain access to the gastroplasty. After a guidewire had been positioned in the gastroplasty, a fully covered metal stent (FCSEMS) was placed in parallel and successfully re-established digestive continuity and excluded the fistula ( Video 1 ).

**Fig. 3:**
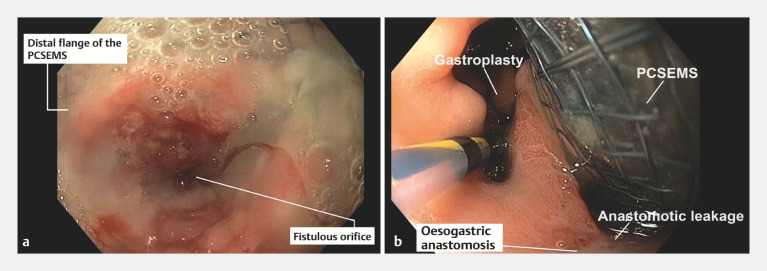
Endoscopic images from the first procedure performed in our center showing: **a** the fistulous orifice and incarceration of the distal flange of the partially covered self-expandable metal stent (PCSEMS); **b** the gastroplasty and passage of the PCSEMS through the fistulous orifice.

Endoscopic procedures are performed firstly to re-establish digestive continuity for optimal nutrition and the anastomotic fistula is excluded by placing a fully covered self-expandable metal stent alongside the partially covered self-expandable metal stent (PCSEMS) that had migrated into the anastomotic fistula; the stent-in-stent technique is subsequently attempted for extraction of the PCSEMS.Video 1

The FCSEMS was removed after 3 months, but the PCSEMS remained irremovable. After discussion with the surgical team, it was decided to try the stent-in-stent technique 5 , and a new FCSEMS was inserted inside the PCSEMS ( Fig. 4 ). A further endoscopy was performed 2 weeks later, at which the FCSEMS was removed without difficulty, but the PCSEMS remained embedded. Given the impossibility of endoscopic PCSEMS removal, it was decided that surgical management would be required and the patient underwent surgical removal of the PCSEMS and coloplasty.

**Fig. 4:**
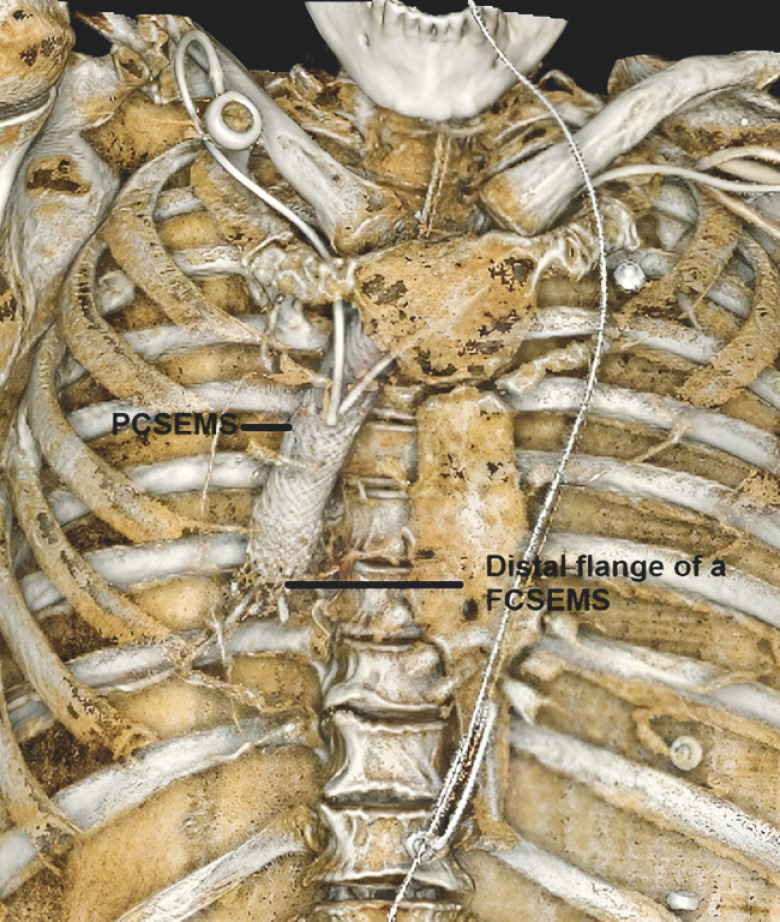
3D reconstruction from the CT scan performed after placement of a fully covered self-expandable metal stent (FCSEMS) within the partially covered self-expandable metal stent (PCSEMS) for the stent-in-stent technique.

Endoscopy_UCTN_Code_TTT_1AO_2AI

## Notes

Endoscopy E-Videos https://eref.thieme.de/e-videos
*E-Videos* is an open access online section of the journal *Endoscopy* , reporting on interesting cases and new techniques in gastroenterological endoscopy. All papers include a high-quality video and are published with a Creative Commons CC-BY license. Endoscopy E-Videos qualify for HINARI discounts and waivers and eligibility is automatically checked during the submission process. We grant 100% waivers to articles whose corresponding authors are based in Group A countries and 50% waivers to those who are based in Group B countries as classified by Research4Life (see: https://www.research4life.org/access/eligibility/ ). This section has its own submission website at https://mc.manuscriptcentral.com/e-videos .
