# Correction: Rescue endoscopic treatment with completion by radical surgery following misplacement of a partially covered metal stent in an anastomotic fistula post-Lewis Santy esophagectomy

**DOI:** 10.1055/a-2408-9537

**Published:** 2024-09-06

**Authors:** Pierre Mayer, Lucile Héroin, François Habersetzer, Pierre-Yves Christmann, Jérôme Huppertz, Leonardo Sosa-Valencia, Abdenor Badaoui

**Affiliations:** 1 36604 Department of Gastroenterology and Hepatology, University Hospitals Strasbourg, Strasbourg, France; 2 560036 Digestive Endoscopy Unit, IHU Strasbourg, Strasbourg, France; 3 27083 Inserm U1110, Université de Strasbourg, Strasbourg, France; 4 Department of Hepatogastroenterology, Clinique Sainte Barbe, Strasbourg, France; 5 560036 Research, IHU Strasbourg, Strasbourg, France; 6 Department of Gastroenterology and Hepatology, CHU UCL Namur, Université catholique de Louvain, Yvoir, Belgium



In the above-mentioned article the affiliation for Abdenor Badaoui has been corrected. Correct is the following institution: Department of Gastroenterology and Hepatology, CHU UCL Namur, Université catholique de Louvain, Yvoir, Belgium. This was corrected in the online version on September 6, 2024.

